# Diagnosis of abdominal tuberculosis: lessons learned over 30 years: pectoral assay

**DOI:** 10.1186/s13017-019-0252-3

**Published:** 2019-07-12

**Authors:** Fikri M. Abu-Zidan, Mohamud Sheek-Hussein

**Affiliations:** 1 Department of Surgery, College of Medicine and Health Sciences, UAE University, Al-Ain, 17666 United Arab Emirates; 2 Institute of Public Health, College of Medicine and Health Sciences, UAE University, Al-Ain, 17666 United Arab Emirates

**Keywords:** Tuberculosis, Extrapulmonary, Abdominal, Diagnosis, Surgery, Algorithm

## Abstract

Diagnosing abdominal tuberculosis remains a great challenge even for experienced clinicians. It is a great mimicker that has unusual presentations. A high index of suspicion is essential for reaching its diagnosis. Clinical and radiological findings of abdominal tuberculosis are non-specific. Herein, we report the lessons we have learned over the last 30 years stemming from our own mistakes in diagnosing abdominal tuberculosis supported by illustrative challenging clinical cases. Furthermore, we report our diagnostic algorithm for abdominal tuberculosis. This diagnostic algorithm will help in reaching the proper diagnosis by histopathology or microbiology. Our diagnostic workup depends on categorizing the clinical and radiological findings of abdominal tuberculosis into five different categories including (1) gastrointestinal, (2) solid organ lesions, (3) lymphadenopathy, (4) wet peritonitis, and (5) dry/fixed peritonitis. The diagnosis in gastrointestinal tuberculosis and dry peritonitis can be reached by endoscopy. The diagnosis in solid organ lesions can be reached by ultrasound-guided aspiration. The diagnosis in wet peritonitis and lymphadenopathy can be reached by ultrasound-guided aspiration followed by laparoscopy if needed. Diagnostic laparotomy should be kept as the last option for achieving a histological diagnosis. Capsule endoscopy and enteroscopy were not included in the diagnostic algorithm because of the limited data of using these modalities in abdominal tuberculosis. They need special expertise, and rarely used in low- and middle-income countries. Furthermore, capsule endoscopy may cause complete intestinal obstruction in small bowel strictures. A definite diagnosis can be reached in only 80% of the patients. Therapeutic diagnosis should be tried in the remaining 20%.

## Introduction

Charles Dickens (1812–1870) has described tuberculosis (TB) as “a dread disease in which struggle between soul and body is gradual quiet and solemn, that day by day, and grain by grain, the mortal part wastes and withers away.” This may be true till now. Tuberculosis is one of the top 10 causes of death, globally. In 2017, ten million people developed tuberculosis, with an estimated 1.3 million deaths [1]. Furthermore, about one-quarter of the global population has latent tuberculosis infection [2]. Currently, the management is even more complex with the emerging of multi drug-resistant bacteria.

Extrapulmonary tuberculosis occurs in about 20% of tuberculosis [3] while abdominal tuberculosis constitutes about 10% of extra-pulmonary tuberculosis [4]. There are three ways in which the tubercle bacilli can infect the abdomen: (1) through ingestion of infected sputum or milk, (2) through hematogenous or lymphatic spread and finally (3) through direct spread into the peritoneum from the fallopian tubes [4, 5]. Surgery is performed in about 15% of the cases of abdominal tuberculosis; half of these are performed as acute surgery including obstruction, abscess formation, perforation, or hemorrhage with the other half as a diagnostic procedure [6]. We have treated 24 cases of proven abdominal tuberculosis in Al-Ain Hospital, Al-Ain, United Arab Emirates, during the last 8 years with an average of 3 new cases every year in a hospital covering a population of 600,000. That would be less than 1% of the acute abdomen admitted to our hospital and gives an incidence of abdominal tuberculosis of about 0.5 per each 100,000 population per year in our current setting. In comparison, 44 cases of abdominal tuberculosis were treated in Mubarak Al-Kabeer and Adan Hospitals, Kuwait, during the period of 1981–1990 which was covering a population of 1,250,000, giving an incidence of 0.35 per each 100,000 population per year. Eight of these 44 patients had pulmonary tuberculosis (18%), 2 had soft tissue tuberculosis (4.5%), 1 had spinal tuberculosis (2.3%), 1 had a brain tuberculoma (2.3%), and 1 had tuberculous cervical adenopathy (2.3%) (Abu-Zidan FM. Management of abdominal tuberculosis in the Gulf region. Unpublished data).

The diagnosis of abdominal tuberculosis remains one of the most challenging tasks in clinical practice. With increased immigration and increased HIV, clinicians worldwide are faced more and more with such unfamiliar cases. We have observed that the common misconceptions on abdominal tuberculosis did not change over the last 30 years. These misconceptions are (1) abdominal tuberculosis is rare, (2) abdominal tuberculosis is always associated with active pulmonary tuberculosis, and (3) abdominal tuberculosis is a disease of the poor [7]. These misconceptions usually distract experienced clinicians from reaching the proper diagnosis.

**Fig. 1:**
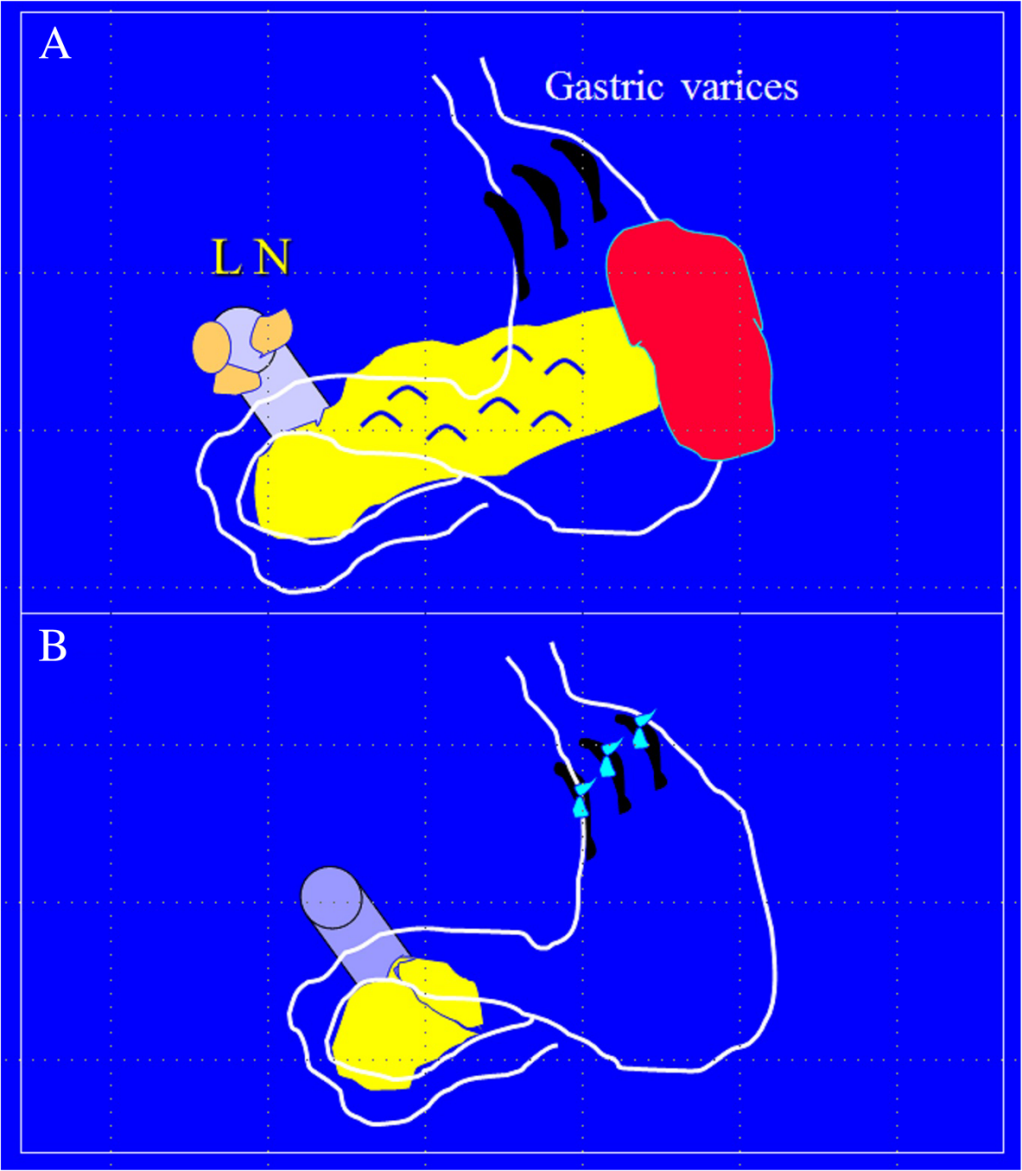
A 23-year-old man presented with severe hematemesis due to gastric varices. The patient had a laparotomy. There was a matted mass in the pancreatic region and lymph nodes compressing on the portal vein mimicking a pancreatic cancer (**a**). The patient had major surgery including distal pancreatectomy, splenectomy, removal of the lymph nodes at the porta hepatis, and suture ligation of the varices (**b**). Histopathology confirmed the diagnosis of abdominal tuberculosis. (Illustrated by Professor Fikri Abu-Zidan, Department of Surgery, College of Medicine and Health Sciences, UAE University). The full clinical details of this patient have been published before [8]

The first author of this manuscript (FAZ) reported an unusual case of abdominal tuberculosis in 1990 [8]. A 23-year-old man presented with severe hematemesis caused by gastric varices due to lymph nodes compressing on the portal vein (Fig. 1a). The patient had a laparotomy. There was a matted mass in the pancreatic region mimicking a pancreatic tumor. Intraoperative frozen section was non-conclusive. The patient had major surgery including distal pancreatectomy, splenectomy, removal of the lymph nodes at the porta hepatis, and suture ligation of the varices (Fig. 1b). It was an unexpected surprise to find that histopathology of the lymph nodes was diagnostic of abdominal tuberculosis. The patient had anti-tuberculous treatment. After 18 months, follow-up computed tomography (CT) scan and endoscopy were normal. This patient could have possibly been treated medically if the diagnosis was reached before surgery. Abdominal tuberculosis is essentially a medical disease and surgical interventions should be reserved for complications including obstruction, perforation, fistulation, or bleeding [4, 5, 9].

This unusual dramatic presentation stimulated great interest in this challenging diagnosis. We will try in this communication to highlight the important lessons we have learned over the last 30 years. Due to increased immigration, we think that these lessons are important and will be useful for young surgeons who may not have faced abdominal tuberculosis before especially in developed countries. We will try to highlight each lesson by an illustrative clinical case to support our statements. Finally, we will describe an algorithm for diagnosing abdominal tuberculosis that was developed over the years and that can be useful globally including low- and middle-income countries.

### Lesson 1: Abdominal tuberculosis is a great mimicker

The lesson learnt from the first case (Fig. 1) is that abdominal tuberculosis is a great mimicker [5, 9]. This is because it can affect single abdominal organs without chest involvement. Other organs are usually not involved. A high index of suspicion is needed for this diagnosis [5, 9, 10]. We have personally encountered cases in which isolated single organ abdominal tuberculosis mimicked pancreatic tumors, colonic cancer, gastric cancer, and lymphomas. It can also mimic infectious diseases including appendicitis, acute cholecystitis, typhoid fever, and necrotizing fasciitis [11–14]. Even in areas where the disease is prevalent, a correct clinical diagnosis is made in only half of the patients [15]. Malignancy was the preoperative diagnosis in 25% in our own series [16].

### Lesson 2: Radiological finding of abdominal TB is non-specific

Ultrasonography and computed tomography CT scan may show generalized or localized ascites with thin mobile septa, thick omentum and peritoneum, lymphadenopathy, or thickened bowel [4, 17–19]. CT scan is the modality of choice in evaluating the extent and type of abdominal tuberculosis [4, 5, 10, 20, 21]. Nevertheless, radiological findings are non-specific [22] and a microbiological or histopathological confirmation should be obtained by percutaneous aspiration or direct biopsy [18].

### Lesson 3: CT scan can miss liver tuberculosis

**Fig. 2:**
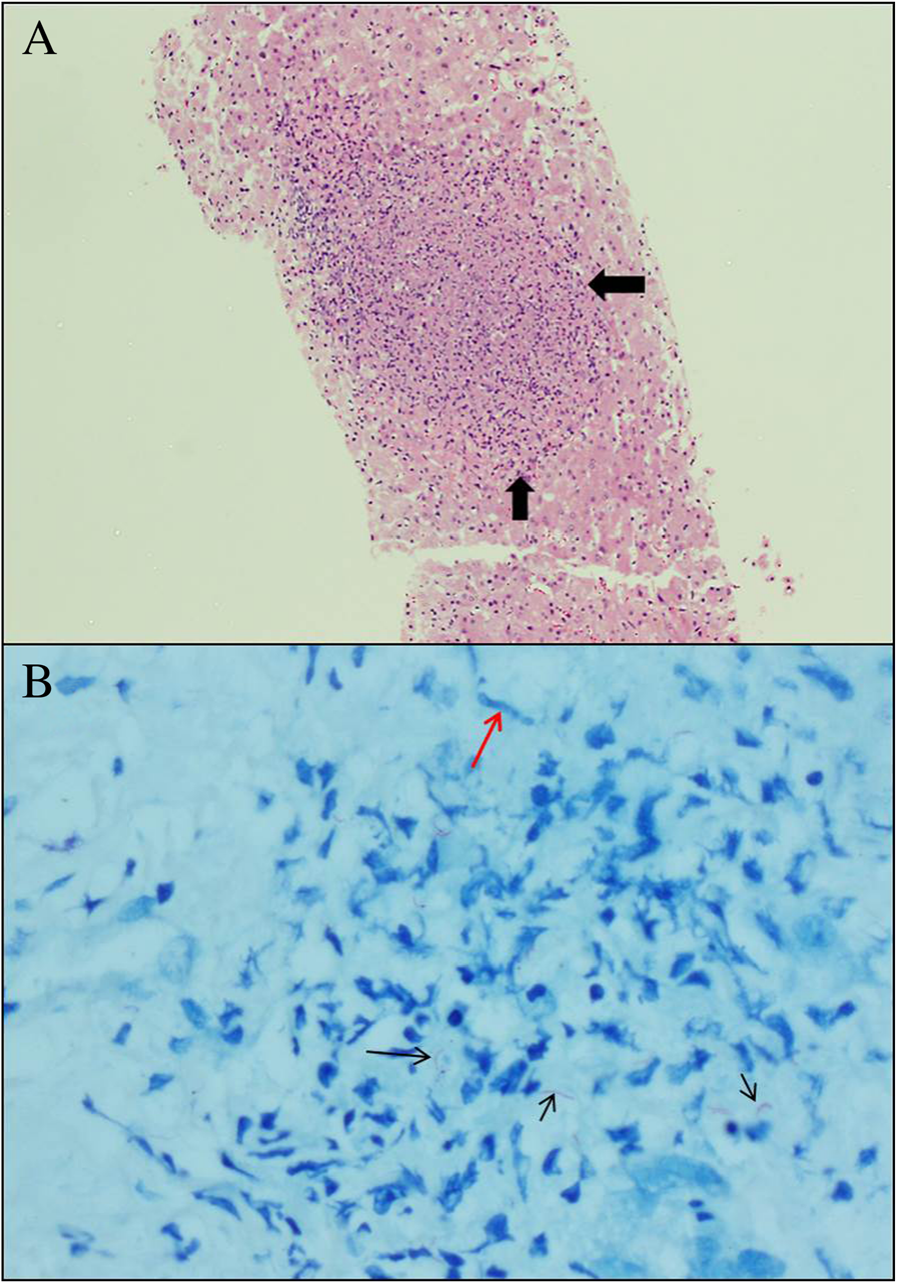
A 39-year-old African man had renal transplant 3 months before presenting to the hospital with an unexplained high fever. His organ functions deteriorated quickly and he was admitted to the ICU with severe sepsis. He needed assisted ventilation, his renal function deteriorated quickly, and his bilirubin and liver enzymes became very high. The patient did not respond to empirical antibiotics. Abdominal CT scan showed a normal liver and spleen with increased enhancement without focal lesions. Tuberculosis was suspected because of a previous history of exposure to tuberculosis despite the negative CT findings. Liver biopsy was performed which was diagnostic of TB. **a** Hematoxylin and Eosin (× 4), showed a well-circumscribed granuloma (arrows) within liver tissue, without evidence of caseous necrosis or giant cells. **b** Ziehl-Neelsen stain ((× 40), for mycobacterium tuberculosis revealed numerous red rods or bacilli (black arrows). In addition, epithelioid macrophages (red arrow) and lymphocytes were identified (Courtesy of Navidul Haq Khan, Consultant pathologist, Tawam Hospital, Al-Ain, UAE)

Normal abdominal CT scan does not rule out hepatic tuberculosis. Small granulomas of military hepatic TB can be missed by CT scan [20, 22, 23] and may be evident only on a biopsy (Fig. 2). If there is high suspicion of hepatic tuberculosis, with raised bilirubin, especially in unexplained severe sepsis not responding to empirical antibiotics in an endemic area of tuberculosis, a liver biopsy is advised, even if the ultrasound and CT scan of the liver are normal**.**

### Lesson 4: An algorithm to diagnose abdominal tuberculosis

**Fig. 3:**
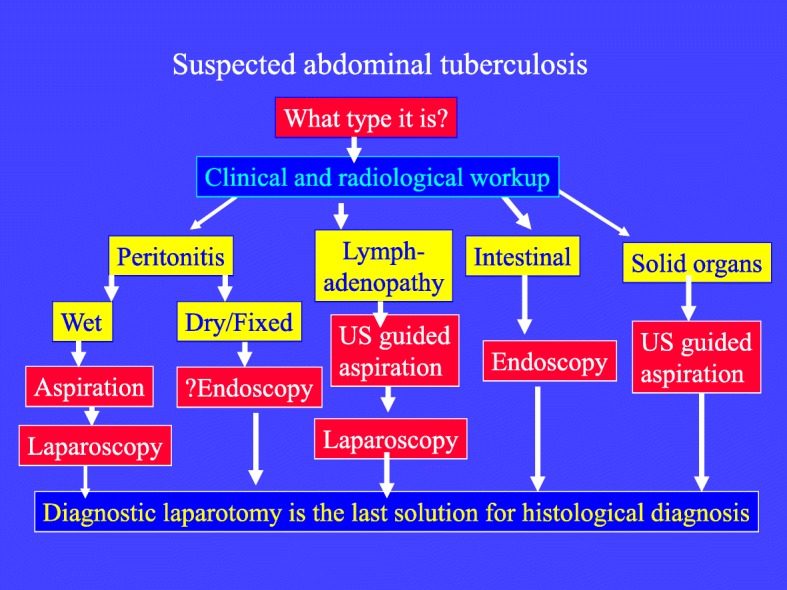
The diagnostic algorithm of abdominal tuberculosis depends on categorizing the clinical and radiological findings into five different categories including (1) gastrointestinal, (2) solid organ lesions, (3) lymphadenopathy, (4) wet peritonitis, or (5) dry/plastic peritonitis

Clinical and radiological findings of abdominal tuberculosis are non-pathognomonic. Culture results may take up to 6 weeks to be reported. So we should aim at getting an early histopathological diagnosis to start treatment [9]. Our diagnostic workup depends on categorizing the clinical and radiological findings of abdominal tuberculosis into five different categories including (1) gastrointestinal, (2) solid organ lesions, (3) lymphadenopathy, (4) wet peritonitis, and (5) dry/fixed peritonitis [4, 5] (Fig. 3). The diagnosis in gastrointestinal tuberculosis and dry peritonitis could be reached by endoscopy and biopsy. The diagnostic accuracy will increase with increased biopsies [4, 10, 24]. Biopsies taken by colonoscopy in 50 patients of colonic tuberculosis were diagnostic in 40 (80%) [24]. The diagnosis of solid organ lesions could be reached by ultrasound-guided aspiration [25–27]. The diagnosis in wet peritonitis and lymphadenopathy could be reached by ultrasound-guided aspiration followed by laparoscopy if needed [28–30]. Diagnostic laparotomy should be kept as the last option for reaching a histological diagnosis.

We did not include capsule endoscopy and enteroscopy in the diagnostic algorithm because of the limited data of using these modalities in abdominal tuberculosis [4]. We did not use this modality in abdominal tuberculosis in our setting. Furthermore, it is expensive, needs special expertise, and rarely used in low- and middle-income countries. Furthermore, capsule endoscopy may cause complete intestinal obstruction in small bowel strictures.

### Lesson 5: Beware of laparoscopy in fibrotic-fixed peritonitis

**Fig. 4:**
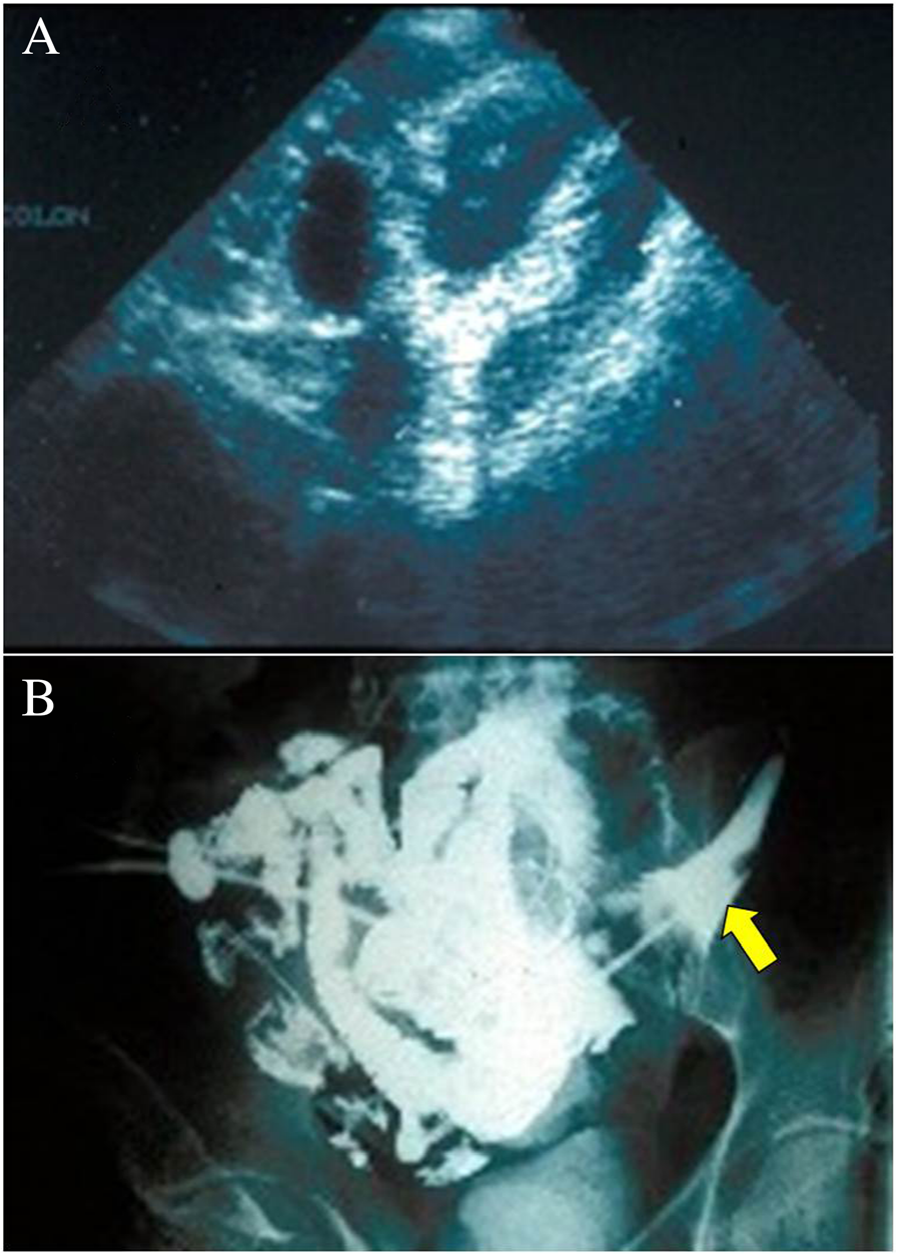
A 50-year-old man presented with abdominal pain of 1 year duration and weight loss. Abdominal examination revealed an abdominal mass in the left lower quadrant. The patient had anemia (hemoglobin of 87 gm/L) and hypoalbuminemia (28 g/L). Abdominal ultrasound (**a**) showed matted bowel loops, thickened mesentery, and presence of intraperitoneal fluid. CT abdomen showed thickened intestine with localized ascites and retroperitoneal small lymph nodes. Diagnostic laparoscopy was tried to harvest a biopsy (**b**). It was difficult and a perforation of the small bowel was suspected. Laparotomy was performed which showed that the small bowel was matted. Intraoperative frozen section confirmed the diagnosis of abdominal tuberculosis. Two iatrogenic small bowel perforations were closed using absorbable sutures. The patient developed postoperative small bowel fistula (yellow arrow)

There are mainly three types of tuberculous peritonitis: (1) the *wet type* which is the most common and occurs in 90% of the cases (free ascites or localized fluid), (2) the *dry type* (plastic) having peritoneal nodules and dense adhesions, and (3) the *fibrotic-fixed type* which shows clumping matted bowel loops with thickened mesentery and omentum [4, 19, 31]. Laparoscopy is now used more often for diagnosing tuberculous peritonitis [9]. Nevertheless, we think that it is contraindicated in fibrotic-fixed type due to the high risk of iatrogenic bowel injury and fistula formation because there may be no space to insert the laparoscope. Laparotomy may be indicated in this condition if biopsy is needed (Fig. 4). This point is more important in the cases of the tuberculous abdominal cocoon which is mainly diagnosed intra-operatively. This condition needs open surgery to peel away the fibrous tissue encasing the bowel [32]. Nevertheless, the final decision for laparoscopy will depend on the laparoscopic experience of the surgeon and his/her familiarity with abdominal tuberculosis.

### Lesson 6: The value of therapeutic diagnosis

**Fig. 5:**
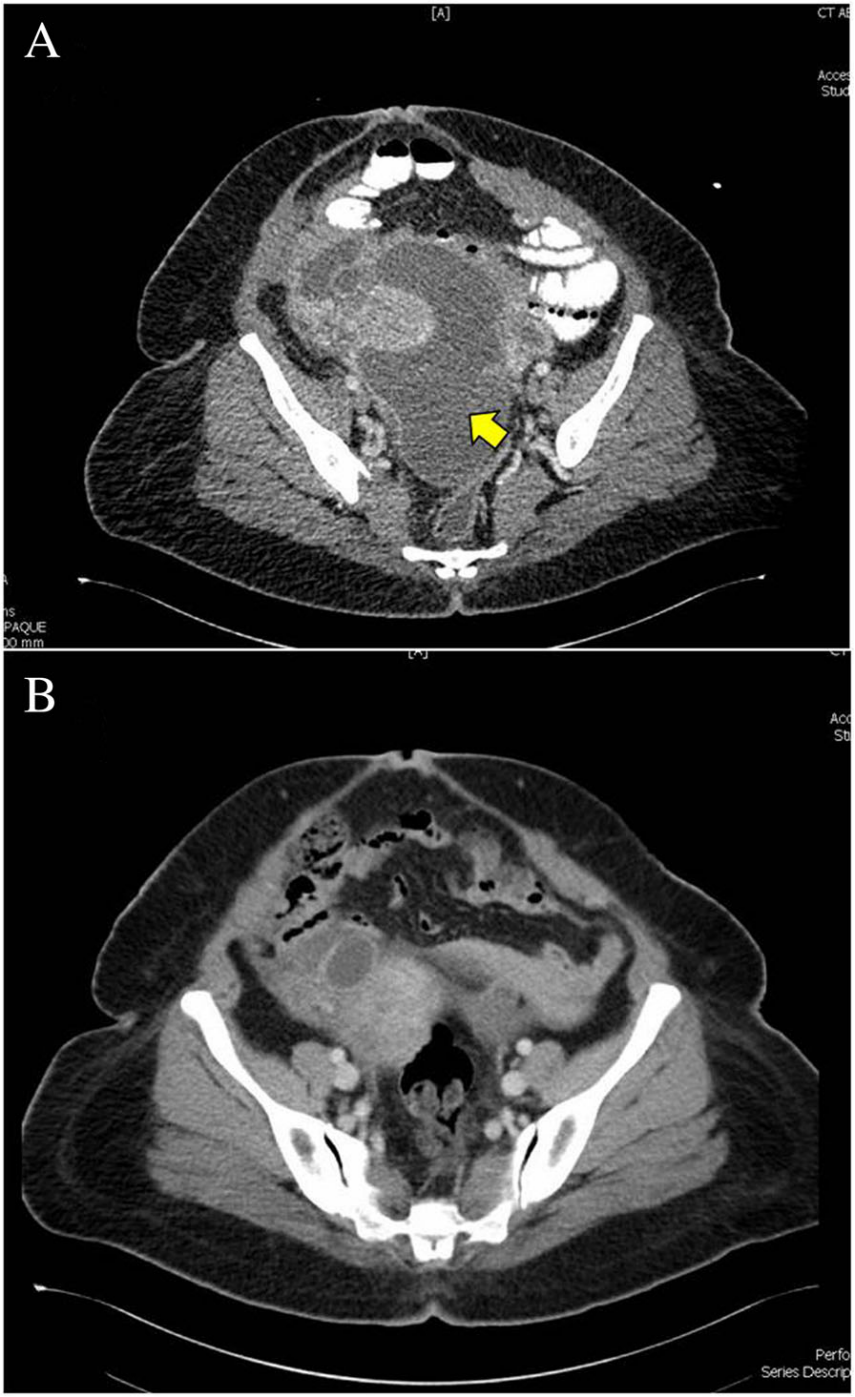
A 44-year-old woman presented with abdominal pain of 3 days duration. The abdomen was distended, tender, but soft. The patients had fever, leukocytosis, and raised C-reactive protein. Abdominal CT scan (**a**) showed multiple intra-abdominal fluid collections (yellow arrow). Green pus was aspirated under ultrasound guidance. The pus culture was negative, and the quantiferon-TB test was undetermined. Abdominal tuberculosis was suspected. A therapeutic diagnosis was successful and the size of the abscess dramatically reduced after 2 months (**b**)**.** (Courtesy of Dr. Hussam Mousa, Consultant General Surgeon, Al-Ain Hospital, Al-Ain, UAE)

Therapeutic diagnosis in different series varied between 16 and 29% [16, 24, 33, 34]. Figure 5 illustrates an example of a therapeutic diagnosis. Although the laboratory results were non-conclusive and the radiological findings were non-specific in this patient, the diagnosis of tuberculosis was suspected and a therapeutic diagnosis was reached. Definite diagnosis can be reached in only 80% of the patients. Therapeutic diagnosis should be tried in the remaining 20%. The majority will have a rapid response to anti-TB treatment, usually within 2 weeks [4].

### Lesson 7: Beware of misdiagnosing TB as Crohn’s disease

Starting patients who have abdominal tuberculosis on steroids on the assumption that it is Crohn’s disease can have dramatic effects and can even lead to death [4, 9]. The differential diagnosis between the two is difficult and there should be every effort to reach a proper diagnosis by getting microbiological or histopathological evidence. The prevalence of the disease in a setting should be considered and caution should be adopted before starting steroids. If in doubt, it may be wiser to start a therapeutic trial of anti-tuberculous treatment as a diagnostic method before steroids.

### The value of new laboratory investigation in abdominal TB

Recently, there have been new immunological and molecular diagnostic techniques for tuberculosis. Nevertheless, a simple global and cost-effective diagnostic laboratory test that can be used routinely to diagnose extra-pulmonary tuberculosis on a global level is still awaited. One of the major limitations of using these new techniques is its cost [35]. We have to be careful when interpreting the published data. Although the sensitivity and specificity of certain tests are very high, the positive and negative predictive values are the important clinical useful values and will change with the prior prevalence of the disease. Furthermore, they do not replace the need for routine AFB smear and culture [36]. Accordingly, the WHO policy recommendation states that “Neither interferon-gamma release assays (IGRA) nor the tuberculin skin test (TST) should be used for the diagnosis of active TB disease in low- and middle-income countries” [37]. Actually, IGRAs are more expensive and more difficult to perform compared with TST although they give comparable results.

When routine laboratory and microbiology tests are non-conclusive, then molecular biology-polymerase chain reaction (PCR) results may support the clinical diagnosis while waiting for the culture results and drug susceptibility [36]. Nevertheless, PCR cannot differentiate between living and dead M. tuberculosis [36, 38]. They remain positive for long periods after completion of anti-TB treatment and death of the bacteria. They should be used only for initial diagnosis and not for follow-up [36]. Furthermore, the excellent results reported from research labs may not be reproduced by service clinical labs. There are contamination, technical, and sampling errors in the clinical labs that may give false positive results and reduce the generalizability of these tests [36].

WHO currently recommends only the Xpert® MTB/RIF assay for diagnosing TB. It can provide results within 2 h [39]. A recent meta-analysis has shown that Xpert has high specificity but limited sensitivity for detecting extrapulmonary TB. A positive Xpert result may rapidly identify TB cases. Nevertheless, negative results cannot rule out the disease [40].

## Conclusions

Tuberculosis is a global health problem. Acute care surgeons should be familiar with the challenges encountered in diagnosing abdominal tuberculosis and try their best to avoid surgery unless indicated [41]. In reality, acute care surgeons may find themselves trapped in situations where peritonitis, unresolved bowel obstruction or suspected bowel ischemia are associated with signs of systemic sepsis that cannot be explained by the non-specific CT findings. Experienced surgeons may decide for an emergency laparoscopy or laparotomy and get surprised by the operative and pathological findings confirming abdominal tuberculosis. A simple cost-effective diagnostic laboratory test that can be used routinely for abdominal tuberculosis is not yet available. Currently, the diagnosis of abdominal tuberculosis should be reached by a combination of clinical, laboratory, radiographic, and pathological findings. A high index of suspicion is essential for reaching this diagnosis. We have shared our mistakes and suggested our diagnostic algorithm for abdominal tuberculosis that was developed over the years hoping that it will be useful for acute care surgeons.

## Data Availability

Not applicable

## Abbreviations

CT: Computer tomography
IGRA: Interferon-gamma release assay
PCR: Polymerase chain reaction
TB: Tuberculosis
TST: Tuberculin skin test
WHO: World Health Organization
