# Correction to: Diagnosis of abdominal tuberculosis: lessons learned over 30 years: pectoral assay

**DOI:** 10.1186/s13017-019-0260-3

**Published:** 2019-08-16

**Authors:** Fikri M. Abu-Zidan, Mohamud Sheek-Hussein

**Affiliations:** 1 Department of Surgery, College of Medicine and Health Sciences, UAE, University, Al-Ain, 17666 United Arab Emirates; 2 Institute of Public Health, College of Medicine and Health Sciences, UAE University, Al-Ain, 17666 United Arab Emirates

**Correction to: World J Emerg Surg. 2019;14:33**

**https://doi.org/10.1186/s13017-019-0252-3**

The original article [1] contains an error in the title - the motif ‘pectoral assay’ should not be present and as such, the title should instead be: ‘Diagnosis of abdominal tuberculosis: lessons learned over 30 years’.
